# P-1356. Cross Sectional Audit and Prospective Study of AWaRe Antibiotics in Three Tertiary Care Hospitals

**DOI:** 10.1093/ofid/ofaf695.1543

**Published:** 2026-01-11

**Authors:** Shibi Selvaraj, R Sanjai, Shobana Selvaganesan

**Affiliations:** Jaya college of paramedical sciences, Chennai, Tamil Nadu, India; Jaya college of paramedical sciences, Chennai, Tamil Nadu, India; Jaya college of paramedical sciences, Chennai, Tamil Nadu, India

## Abstract

**Background:**

Antibiotic resistance poses a significant threat to public health, exacerbated by inappropriate antibiotic use in tertiary care hospitals. A study in three such hospitals can assess stakeholders' knowledge and practices, guiding interventions to enhance antibiotic stewardship. Improved awareness and responsible antibiotic use are crucial in combating resistance and preserving antibiotic efficacy.

**Methods:**

AWaRe has been classified into three groups by WHO – world health organization. They are Access, watch & reserve. A cross sectional prospective study was conducted among three hospitals. The audit tool, a simple and validated instrument, encompassed patient demographics, diagnosis, antibiotic details, infectious disease consultation, final diagnosis. The antibiotic stewardship team, guided by an Infectious Disease specialist, systematically collected and analysed the data using descriptive statistics.

**Results:**

In the study (N=376), 43% received aware antibiotics in three tertiary care hospitals. 18% received access antibiotic; 84% received watch antibiotic and 12% received reserve antibiotic shown in table 1 below

**Table: 1:** AWaRe antibiotic utilization in tertiary care hospitals

CATEGORY	FREQUENCY (N)	PERCENTAGE (%)
Patients Admitted (Total)	376	100%
Patients Prescribed with AWaRe Antibiotic	163	43%
Access	30	18%
Watch	137	84%
Reserve	20	12%

**Conclusion:**

The study reveals concerning antibiotic usage trends across three tertiary care hospitals, with 43% of patients receiving antibiotics, predominantly categorized as Watch (84%). Urgent interventions, including robust stewardship programs guided by infectious disease specialists, are imperative to mitigate resistance emergence and safeguard antibiotic efficacy through enhanced awareness and adherence to guidelines.

**Disclosures:**

All Authors: No reported disclosures

